# Quinto Tiberio Angelerio and New Measures for Controlling Plague in 16th-Century Alghero, Sardinia

**DOI:** 10.3201/eid1909.130311

**Published:** 2013-09

**Authors:** Raffaella Bianucci, Ole Jørgen Benedictow, Gino Fornaciari, Valentina Giuffra

**Affiliations:** University of Turin, Turin, Italy (R. Bianucci);; University of Oslo, Oslo, Norway (O. Jørgen Benedictow);; University of Pisa, Pisa, Italy (R. Bianucci, G. Fornaciari, V. Giuffra)

**Keywords:** Yersinia pestis, Sardinia, 16th-century Sardinian medicine, sanitary measures, bacteria, vector-borne infections, zoonoses, plague

## Abstract

Plague, a zoonotic disease caused by the bacterium *Yersinia pestis*, has been responsible for at least 3 pandemics. During 1582–1583, a plague outbreak devastated the seaport of Alghero in Sardinia. By analyzing contemporary medical texts and local documentation, we uncovered the pivotal role played by the *Protomedicus* of Alghero, Quinto Tiberio Angelerio (1532–1617), in controlling the epidemic. Angelerio imposed rules and antiepidemic measures new to the 16th-century sanitary system of Sardinia. Those measures undoubtedly spared the surrounding districts from the spread of the contagion. Angelerio seems to have been an extremely successful public health officer in the history of plague epidemics in Sardinia.

The Black Death, a huge wave of epidemics of bubonic plague, spread across Europe during 1347–1353 CE. As detailed in nearly 200 local mortality studies relating to southern and western Europe, at least half of the population died of plague (*1*). The Black Death (1346–1353 CE) was the first outbreak of the second plague pandemic that occurred repeatedly until 1750 CE. Most likely it originated in the old plague reservoir (i.e., wild rodents) stretching from the northwestern shores of the Caspian Sea into southern Russia. Russian and Byzantine chroniclers mention the outbreak of terrible diseases there in spring 1346.

Kaffa, the far-outlying Italian trading station in Crimea, also was a source of infection. During spring 1347, Italian galleys fleeing Kaffa brought infection to Constantinople, where the plague began raging in the summer. From Constantinople, ships carried plague to ports along the Mediterranean littoral whence the infection fanned out from several epicenters, acquiring new momentum from these new centers as it spread (*1*).

Stimulated by earlier observations that epidemic diseases were transported by ships, the notion of quarantines began being developed in the early 14th century. Accordingly, a genuine quarantine was set up in 1377 in the Venetian trading station at Ragusa (present-day Dubrovnick, Croatia). Thirty-day isolation was imposed for ships from areas that were infected or suspected of being infected and 40-day isolation for land travelers from these areas (*2*,*3*).

During the 15th and 16th centuries, quarantine and sanitary cordons were imposed. Contacts and trades with infected regions were banned, and towns’ gates and states’ frontiers were closed, which prevented free movement of humans and merchandises to avoid the risk of spreading the contagion (*4*).

The first *lazaretto* (plague hospital/infirmary) was set up in 1423 in Venice. This institution soon became a model for isolating infected patients and preventing the spread of the epidemics (*3*). The *lazaretto* reflected the development of epidemiology and increasing administrative skills in Renaissance society.

During 1350–1520, ≈100 plague tracts were published (*5*). They shared a view of epidemic diseases: the final cause was God’s anger over his human subjects’ sins, and epidemic disease was His punishment. The means of punishment, the pathogenic causal agent, was miasma, a notion of Greek Hippocratic–Galenic medicine (*2*,*6*). Miasma was corruption or pollution of the air by noxious vapors containing poisonous elements caused by rotting putrid matter. Medieval medical theory added geophysical and astrologic elements; miasma also could be let out from the ground by volcanic activity or particular constellations of planets. Miasma was spread by wind and therefore could spread speedily; it could enter humans by inhalation or through the pores of the skin.

The theory was that miasma wa the only cause of epidemic disease. The variety of epidemic diseases and their clinical and epidemiologic manifestations were explained by miasma’s ability to evolve into agents with different pathogenic properties, so a mild disease could develop into plague. According to miasmatic theory, plague patients were contaminated by the most dangerous type of miasma; air of the room also was contaminated by it (*6*).

The plague tracts warned all persons, including physicians, not to enter the rooms of plague patients or perform clinical examinations of such patients. Patients should be contacted from a distance. Thus, clinical elements mentioned in plague tracts were not empirical observation but based on hearsay. Some of this hearsay was consistent, especially that plague buboes developed in plague patients; most often these were visible on the neck because patients were not physically examined.

Not until the Renaissance, in the decades around 1500, was the theory of miasma expanded to include the idea that healthy persons could be infected by touching infected persons or objects contaminated by them with miasma (fomites), the *Fracastoro* miasmatic–contagionistic theory of cross-infection and epidemic spread (*1*–*6*). The basic tenets of miasmatic–contagionistic theory governed the actions and epidemic countermeasures of governments and municipal councils and their medical advisers. This theory explains why measures such as quarantines, sanitary cordons, isolation of persons with and suspected to have plague and with objects used by them, disinfection of houses, and disinfection of textiles were implemented beginning in the early 1500s.

Consequently, through special laws, administrative institutions were created to manage the organization of the sanitary system during plague outbreaks. The most efficient system of prevention and control was established in north-central Italy by the cities of Venice, Genoa, Florence, and Milan during the 14th and 15th centuries (*2*–*4*).

However, in Sardinia, early Modern Age (late 14th–early 15th centuries) health systems were considered mediocre (*7*,*8*). From the arrival of the Black Death in Sardinia (in 1348), plague epidemics required day-by-day organization. No social or political measures were pursued to control and prevent the plague outbreaks.

Despite the lack of organization in the Sardinian health system, in 1455, King Alfonso V of Aragon (1396–1458) established and imposed improvements. The royal ordinance created and imposed the so-called Office of the *Protomedicus* of the Sardinian Reign in Cagliari. Although this institution was already operating in the Catalan–Aragonese region, it represented an innovation for the island. The *Protomedicus*, a person belonging to the upper class and possessing a medical degree, superintended the medical practice and selected persons to certify to perform as physicians. Together with the municipal authorities, the *Protomedicus* coordinated prophylaxis and therapies (*8*).

The Catalan–Aragonese sanitary system of the second half of the 15th century had a Health Guard or Plague Guard, also called *Morber.* A *Morber* was also installed in Sardinia at that time (*8*,*9*). The *Morbers*’ task was to watch over the sanitary conditions of the ships docking at the island’s harbors by halting the disembarkation of persons with or suspected to have plague and to assist the *Protomedicus* during the plague outbreaks.

During the 15th and 16th centuries, Sardinian literature that focused on the history of medicine was poor and attests to the extreme backwardness of the medical culture and the sanitary structures (*8*). During the 16th and 17th centuries, only a few qualified physicians, selected by the *Protomedicus*, were practicing in Sardinia (*8*). Because of the absence of local universities, upper class students willing to perform medical studies completed their education in universities in Spain or Italy and seldom returned to Sardinia (*10*). Even after the foundation of the Universities of Sassari (1617) and Cagliari (1626), the situation remained almost unchanged because a rigorous faculty of medicine was still lacking (*10*).

The deficient professional background of the Sardinian physicians was reflected in poor communal organization of the sanitary structures. Those limits affected any attempt to prevent and contain the plague outbreaks that lashed the island from the time of the Black Death onward (*8*,*11*,*12*).

We report on the Neapolitan physician Quinto Tiberio Angelerio (*13*,*14*), *Protomedicus* of Alghero, who provided a breakthrough in the fight against the 1582–1583 plague epidemic by introducing novel prophylactic measures. Angelerio’s scientific background was influenced by Galen’s miasmatic theories and by Fracastoro’s contagionistic theories (*2*,*3*). In addition, Angelerio had experience with plague before coming to Alghero. He had practiced in Messina, Sicily, during the 1575–1576 plague epidemic. At that time (1575), the *Protomedicus* of the Sicilian Reign, Giovanni Filippo Ingrassia (1510–1580), was successfully battling the plague outbreak that was spreading in Palermo (*15*).

Ingrassia had introduced useful prevention measures against plague, which included the isolation of persons with and suspected to have plague and of convalescents in 3 different isolation centers; the disinfection of the houses in which plague-related deaths had occurred; and use of dry heat to eliminate the “seeds of contagion” from everyday objects, thus anticipating the concept of modern sterilization (*15*,*16*).

No historical sources provide evidence of direct contact between the 2 physicians. Nonetheless, Angelerio’s observations in Sicily formed his notions of how to combat such epidemics (*8*). To stem the spread of the contagion, Angelerio established a set of sanitary and prophylactic instructions that showed strong analogies with those previously adopted by Ingrassia (*8*).

## Historical Sources and Demographic Data

**Figure 1:**
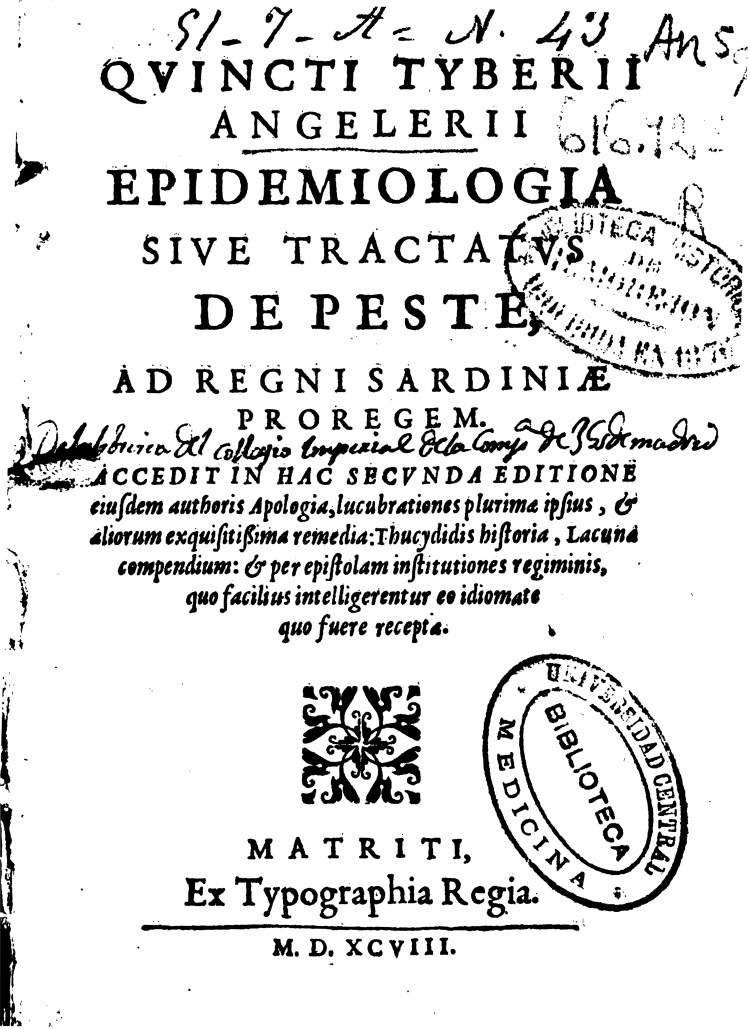
Frontispiece of the *Epidemiologìa Sive Tractatus de Peste*.

Using contemporary documents, we reconstructed the measures introduced by Angelerio and the city government to prevent and control plague epidemics (*17*). The history of the 1582–83 epidemic, which lasted 8 months, is detailed in *Ectypa Pestilentis Status Algheriae Sardiniae* (Instructions on the Alghero, Sardinia, Plague Epidemic) (p. 110) (*17*) (Figure 1**)**. Angelerio wrote and dedicated the booklet, published in 1588 in Cagliari, to the Viceroy De Moncada (*17*,*20*). Two printed versions and a manuscript are extant. The 1588 edition was written in Latin with a 12-page addendum in Catalan entitled *Instructions del Mates Autor* (Instructions from the same author). The second edition, published in Madrid in 1598, was entitled *Epidemiologìa sive Tractatus de Peste* (Epidemiology or Treatise on Plague) and was written exclusively in Castilian (*18*,*20*). A copy of each edition is kept in the University Library of Cagliari, and 1 copy of the Ectypa is preserved at the Alghero’s Municipal Library (*19*). The detailed sanitary measures formulated in Ectypa included 57 instructions (Table 1, Appendix). In the Epidemiologìa, the number of instructions was reduced to 30.

**Table 1:** Sanitary measures described in the original text, *Ectypa Pestilentis Status Algheriae Sardiniae* (1588), by the *Protomedicus* Quinto Tiberio Angelerio (1532–1617)*

No.	Instructions
I	Because the disease is considered a divine punishment, fasts, prayers, vows, and good actions are prescribed to appease the wrath of God.
II	The town must be divided into 10 wards. Each ward must be controlled by a Health Deputy, a person with a high reputation who is invested with full powers. The Health Deputies have the power and the means to 1) punish disobedient citizens without the need to ask for any Magistrate’s advice; 2) set fire to all objects suspected to be infected with plague; 3) close the houses in which plague casualties had occurred; and 4) provide the guards and adopt any mandatory measure needed to guarantee the public health.
III	Through edicts, the population must be warned that citizens who do not declare new plague cases—cases that occur in their houses and in other houses—within 6 hours will be prosecuted.
IV	It is strictly forbidden to have contact with a person suspected to have contracted plague before a physician has ruled out the suspicion.
V	The plague hospital must be kept closed by establishing strict guards, thus avoiding the risk that plague patients will mingle with the rest of the population. All patients will be provided all supplies and medicine needed.
VI	The Health Deputies and the *Morbers* must gather twice a day in the so-called “City House” to follow the course of the epidemic and to transmit the information to the Councilors who are assisted by the physicians.
VII	Fire must be set to mattresses, fittings, and furniture from all houses in which plague cases have been registered.
VIII	If paupers become ill of a “common” disease and do not want to leave their houses, the city government must provide them with the supplies and medicine that are commonly guaranteed in the hospital.
IX	Meetings, dances, and entertainments are strictly forbidden.
X	When a person is suspected to have died of plague, the corpse must be checked by physicians or surgeons to establish whether the deceased person actually died of plague. If the cause of the death is indeed plague, the relatives of the deceased person must carry the corpse in the courtyard or leave it outside the door.
XI	Two secluded infirmaries must be chosen where persons with plague or suspected to have plague can be isolated. Until these sites are assigned, these persons will be allowed to live in their own houses. However, they should keep themselves separate from the rest of their families as much as possible. Guards will watch over their houses.
XII	Gravediggers should be selected from among persons who had contracted and survived plague during a previous outbreak in another town. Gravediggers must live separately from the rest of the community and far from the hospital. They are not allowed to leave their houses unless accompanied by a Health Deputy.
XIII	Furniture and fittings that are not used must be put aside so that they do not get infected; this will occur until the whole city undergoes disinfection.
XIV	The Councilors and the Jurors of the city of Barcelona (with whom the city has many commercial exchanges) must be immediately informed that a plague outbreak is occurring in Alghero.
XV	Selling entrails of old animals, meat from ill animals, pool fishes, and any other kind of low-quality meat is forbidden.
XVI	Each day, the *Morbers* and the physicians are compelled to visit all houses suspected to have plague patients and to arrange the hospitalization of these persons into the isolation center (*tancat*).
XVII	Persons from a house with persons suspected to have plague are forbidden to leave their premises to reach the core of the city. The *Morbers* are charged to fulfill their needs.
XVIII	If plague affects a person living in a house suspected to have persons with plague, gravediggers must take the patient to the hospital or to the isolation place by moving him/her and the bed in which he/she lies. Leaving a plague patient in his/her own house is absolutely forbidden. The transfer to the *tancat* has to take place immediately. If the patient is a distinguished person, he is allowed to stay in his own house.
XIX	A red cross must be painted on the doors of houses known or suspected to have persons with plague so that the rest of the population will keep its distance.
XX	The surgeons are not allowed to leave the hospital or the isolation center. They are allowed to leave those structures only to assist other plague patients, and they must be accompanied by the *Morbers* and the guards.
XXI	Trustworthy persons must be elected to stay in the isolation center and to assist plague patients.
XXII	The pharmacists must provide the poorest with the necessary treatments. A list of the supplied treatments and a list of the citizens must be kept to distinguish between the poorer and the richer. The richer will pay for their treatments, and the city government will pay for the paupers.
XXIII	The city must be cleaned every week from rags and dead things; leather not tanned and rotten raw wool must be put in isolated places; turkeys and cats must be killed and thrown in the sea.
XXIV	It is compulsory to use the Armenian bole for the disinfection of wells and wine casks. Every month, a sack of Armenian bole must be poured in each well. A certain amount also must be added to the wine casks so that they are preserved from the bad quality and corruption of the plague humors.
XXV	A good supply of wood must be provided to light fires in the city and in the houses during the days and nights. Persons must wear perfumes to eliminate or to mitigate the bad quality of the corrupted air.
XXVI	Fire must be set to the infected objects with no peculiar value. High-value furniture must be washed; exposed to the wind; or even better, disinfected in dry heated stoves/ovens.
XXVII	Frequent inventories must be carried out in the pharmacies to guarantee a large stock of medicines and their availability.
XXVIII	Proclamations must be performed to prevent the citizens from going out of their premises and not move from one house to another. It is forbidden to set fire to furniture and fittings without the respective permission. Those not complying with these instructions will be prosecuted.
XXIX	Bells must be rung and cannon balls and artillery fired to purify the air.
XXX	When the physicians diagnose a new plague case, the *Morbers* must be immediately alerted. They will have the custody of the patient and will take care of him/her.
XXXI	It is compulsory to shut the windows and close the doors of all houses when a person with plague or suspected to have plague is taken to the *tancat* or to the *lazaretto* or when a person who died of plague is taken to the cemetery. Perfumes must be worn and bells must be rung so that the citizens pay attention not to contract the bad air (*disaura*) and, hence, the contagion.
XXXII	It is mandatory to bury plague victims within 6 hours after their death. The corpses must be buried in secluded cemeteries. Long and deep trenches must be excavated, and the corpses must be covered with lime to avoid the air corruption and mephitic vapors. It is forbidden to bury plague victims inside the churches. Citizens who die outside the city walls must be buried in secluded areas.
XXXIII	During the Mass, it is highly recommended to be careful when shaking hands in token of peace.
XXXIV	The beggars and the homeless must be kept outside the city walls during the day to reduce as much as possible their contact with other citizens.
XXXV	All citizens are compelled not to leave their houses. Only 1 member per household is allowed to go out for shopping. Permission to go out has to be granted by the *Morber* of the area.
XXXVI	People allowed to go out must bear with them a cane measuring 6 feet long. It is mandatory that people keep this distance from one another.
XXXVII	Physicians are compelled to visit all patients. The richest will pay in due time, and the city Councilors will provide for the poorest.
XXXVIII	A large rail, called *parabanda,* must be positioned in front of the counters in the shops in which meat, bread, wine, and foodstuff are sold so that citizens will keep their distance from the counter itself.
XXXIX	It is mandatory to keep dry stoves/ovens always on. These stoves/ovens are similar to those used to cook the flat tiles (*rejolas*). The oven’s chamber must be filled with infected textiles/objects after those ones have been washed under the *Morbers*’ supervision. The chamber must be constantly heated by an underlying lighted fire.
XL	To allow people to confess, 3-window portable confessionals must be prepared. Two windows are positioned laterally and 1 anteriorly so that the confessor is not reached by the patients’ bad breath. For the confessor’s sake, the confessional must be perfumed and kept locked in a chapel not accessible to the common people. When sacraments are administered, the confessional must be transported by the gravediggers directly at the patient’s bedside and must be taken immediately back to the chapel.
XLI	The weekly Head of the *Morbers* is charged to list all the things entering the *lazaretto* and the *tancat* during the week and to attend to all the patients’ needs. Similarly, the *Morbers* are charged to fulfill the needs of persons suspected to have plague who must stay isolated in their own houses and watched over by the guards.
XLII	The weekly Head of the *Morbers* must keep the inventory of all the beds, fittings, and furniture that enter into the *tancat* and the *lazaretto*. Things showing a good state of preservation will be used to fulfill the patients’ needs, whereas the rest will be burned to avoid the spread of contagion and to avoid robberies.
XLIII	Citizens are forbidden to attempt to cure themselves in their own premises. All ill persons and persons suspected to have plague must be carried to the *tancat* or to the *lazaretto*. Guards must accompany these persons and keep other citizens away during the transfer.
XLIV	All infected textiles and objects from the *lazaretto* must undergo laundry and then disinfection in the dry oven.
XLV	During summertime, bonfires must be set in wooden areas to purify the air taking care not to damage the land’s owners.
XLVI	Infected infants who are orphans or do not have a wet nurse must be bottle fed by using the milk of well-fed goats. For this purpose, goats will be allowed to live inside the *lazaretto*.
XLVII	The buboes of plague patients must be cut open or cauterized. Those who are reluctant must be tied so that surgeons can intervene.
XLVIII	People suspected to be infected and convalescents must undergo quarantine before they are allowed to get back in contact with healthy inhabitants.
XLIX	When the plague epidemic is close to its end, a high number of male and female goats will have to be introduced within the city walls during the night. The animals will be placed inside the houses of the plague patients, and this operation will be repeated for several nights. However, for the population’s sake, the houses also will undergo whitewashing. Whitewashing will be performed by painters who survived the contagion. For the less suspected houses, it is required that the windows be kept wide open and that perfumes be sprayed and all surfaces washed with vinegar.
L	People living in the surroundings are forbidden to enter the city walls unless their health status has been carefully checked and permission from the *Morbers* granted. The *Morbers* are entrusted to check that these persons’ belongings be washed and disinfected in the oven. Once these persons have been proved to be healthy, they will be admitted to live in their new houses but only after their own disinfection. Once they have settled down in their new houses, these persons are compelled to stay isolated from the rest of the population for some days.
LI	The houses’ owners and the lodgers are compelled to disinfect, whitewash, ventilate, and water their residences. In case they do not attend the task, the city government will have to bear the costs.
LII	It is strictly forbidden to sell linen, silk, cotton, and wool textiles without permission from the *Morber* of the area.
LIII	The *Morbers* are compelled to carry out the complete disinfection of the city afterward and house after house. The darkest houses and those lacking aeration will be whitewashed, perfumed, and cleaned with vinegar. Bonfires will be set all around. Similar precautions will be applied to the houses whose walls are covered with golden slivers and leathers. Silk, cotton, linen, and woolly textiles must be washed and disinfected in the oven.
LIV	Once all these precautions have been taken, the Councilors, the Deputies, the hospital attendants, and the physician Angelerio himself [the other anonymous physician had already died of the plague] must visit all the Alghero inhabitants, house after house. The citizens will be asked under oath if the *Morbers* had indeed disinfected their houses properly. In case disinfection had not properly been performed, another will be carried out within the following 6 days.
LXV	It is compulsory that all citizens expose to the wind the furniture and fittings from their houses for 10 days. When the Counselors visit the citizens, at the end of the epidemic, everything must be in order.
LXVI	Each citizen who is aware that his neighbors have not carried out the disinfection properly must notify it to the Councilors and *Morbers*. The latter will keep the secret and will pay the honest citizen with a money consideration.
LXVII	It is compulsory to disinfect the *tancat* and the *lazaretto* located outside the city walls by using the same methods described in Instruction LIII. Fire must be set to every object kept inside the above structures. The persons who have survived and complied with the quarantine will be allowed to return to the city wearing new and disinfected clothes. The main city hospital, Sant’ Antonio, must be disinfected and reordered. The hospital will be reopened to all the population and will go back to its standard use.

*Morbers, the health or plague guardians. The *Morbers*’ task was to watch over the sanitary conditions of the ships docking at the island’s harbors and assist the *Protomedicus* during the plague outbreaks. The usual *Morbers*’ duties were largely extended during the 1582–1583 plague outbreak; *tancat*, isolation center for persons suspected of having plague; *lazaretto*, Isolation center for plague patients; Armenian bole, a ferruginous, ochreish, red clay used as a therapeutic substance against plague and all types of poisons.

The paucity of contemporary documentary records and the discrepancies between their information made a death rate difficult to estimate (*20*–*25*). Death registers were kept only from 1677 onward (*21*–*23*).

The archival data presented below are from notepads belonging to the Archives of the Diocesan Curia of Alghero (ACVA Battesimi) (*21*–*23*) (Table 2). Because of the absence of burial registers for this period and a nearly 4-year gap (November 26, 1581–November 10, 1584) in marriage registers, the demographic reconstruction of the population’s profile has to be based only on baptismal registers (*21*,*23*). In 1582, a total of 158 children were baptized (Table 2). When we account for the annual mean of 154 baptisms in the 5 previous years (1577–1581) and the fact that the effects of the plague epidemic on the population can be estimated only for the following year (1583), the monthly distribution of baptisms in 1582 appears to be normal: unaffected by plague (*21*,*23*).

**Table 2:** Monthly number of births according to the baptismal registers, Sardinia, 1582–1584

Month	No. baptisms, by year		
	1582	1583	1584
Jan	10	9	7
Feb	12	13	11
Mar	8	5	17
Apr	16	3	15
May	20	2	20
Jun	6	0	11
Jul	12	6	16
Aug	13	6	23
Sep	21	7	20
Oct	15	2	16
Nov	17	4	7
Dec	8	5	10
Total	158	62	173


In 1583, the number of baptisms fell by 59.7% to 62. In 1584, the number of baptisms increased sharply to 173, 12.3% above the average for 1577–1581, indicating the immediate onset of recuperative population growth in the wake of the plague epidemic. This growth resulted from a strong increase in marriages and consequent increase in conceptions and births because young adults could easily find jobs and housing vacated because of the plague and, for the same reason, immigration by young adults into the town from the surrounding countryside (*1*,*6*,*21*,*23*–*26*).

The bibliographer and diplomat Toda y Güell (*27*) claimed that the 1582–1583 outbreak caused 6.000 deaths, and only 150 (*cent y sinquanta*) persons survived.According to modern demographists, it is unlikely that Alghero counted 6.000 inhabitants at the end of the 16th century (*19*,*21*,*22*). Data from the 1589 census (*24*) attribute to Alghero 768 fires (each fire was formed by a family of 4.5–5 persons), which correspond to ≈3,400–3,800 inhabitants. Therefore, it appears that the death rate (97.6%) was intentionally augmented by local authorities to obtain a tax reduction (*14*,*21*,*22*).

## Findings and Discussion

**Figure 2:**
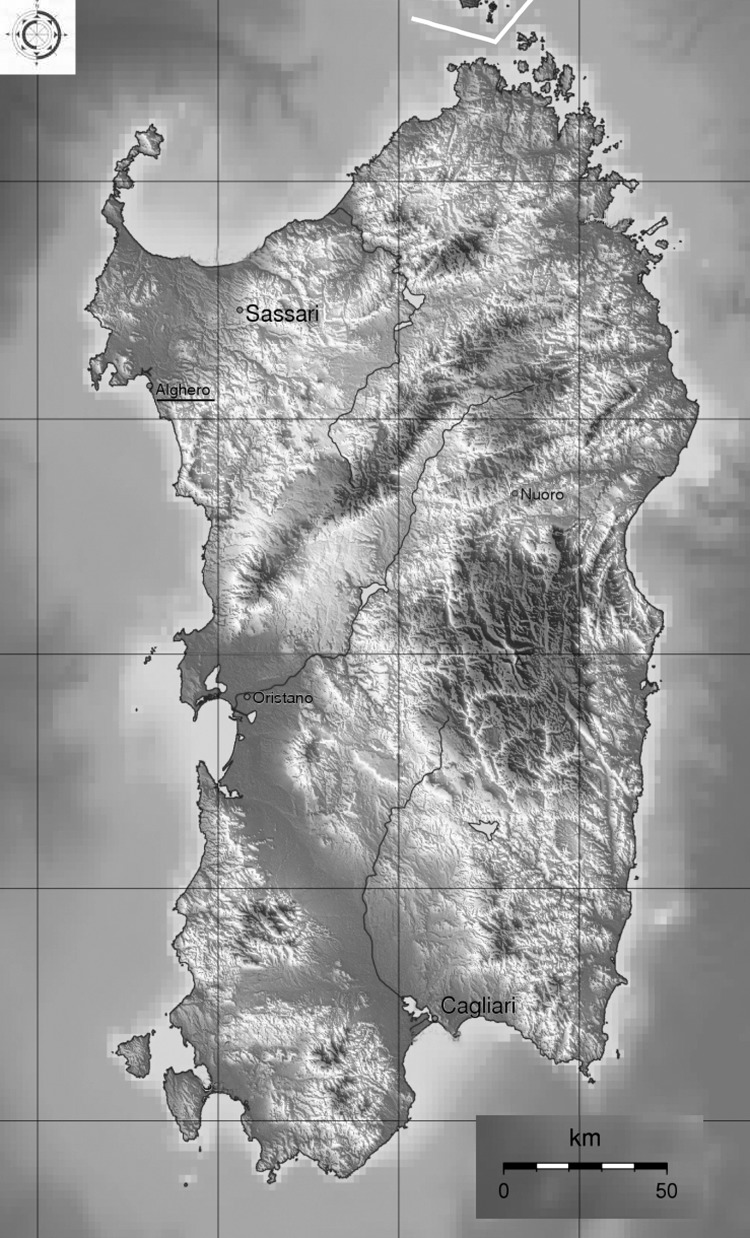
Modern-day Sardinia.

After the 1528–1529 outbreak, Alghero remained free of plague for ≈60 years (*13*,*14*) (Figure 2). The first new casualty attributed to plague in Alghero was registered on November 19, 1582 (*17*,*19*–*22*). The epidemic lasted 8 months and ended in June. After June 14, 1583, no new cases of contagion were registered (*17*).

The *Protomedicus* Angelerio immediately recognized the clinical manifestation of the infection (inguinal buboes and delirium) in a sailor who had disembarked from a ship docked during 1 night at the beginning of November 1582 (*17*). A sailor on a ship from Barcelona was thought to have been the initial harbinger of the infection (*17*). At that time, however, plague was almost absent from Spain while ravaging France. More likely the sailor disembarked from a ship coming from Marseille, where the plague had raged since 1581 (*19*). The *Morbers* had not been effective in halting the arrival of the plague (*9*).

After the sailor’s death, 2 women, a widow named Cifra and her mother, Grazia, who was assisting her at Alghero’s Sant’Antonio Hospital, died in succession. With the help of a priest, Angelerio tried to convince the Bishop, Andrea Baccallar, and the Magistrates to contain the focus of the contagion because he recognized the small punctuate bruises (petecchiae) in the 2 deceased women (*17*,*19*). The Magistrates were indecisive, however, and on the morning of November 20, 1582, Bishop Baccallar asked the Senate to isolate the ill patients. However, the senators did not take Angelerio’s report seriously, and Angelerio was accused of having an apocalyptic vision of the future (*19*). Meanwhile, the son of the widow Cifra died from plague. His death was followed by those of a crippled woman, an old woman, and a young daughter of the widow Crippa.

Because Angelerio was unable to persuade the Magistrates, he turned to the Viceroy, don Michele De Moncada (*8*,*17*). Angelerio explained that the forthcoming plague outbreak would have devastating effects on the population. Furthermore, he detailed the rules and sanitary measures needed to contain the epidemic.

Convinced by Angelerio’s arguments, the Viceroy De Moncada gave orders to block all commerce from and to Alghero. A triple sanitary cordon was established, and triple barriers were built around Alghero’s boundaries. Horse guards checked the city walls (*8*).

The cessation of commerce was taken badly by the inhabitants. Angelerio was loathed by the population, who wanted to lynch him. However, when the contagion spread from the core of the old town to the whole city, Angelerio was finally entrusted with the task of containing the epidemic (*8*,*17*).

Angelerio pioneered the implementation of successful public health measures in 16th-century Sardinia, basing his policies on daily reports of the Alghero population’s health conditions and the incidence and location of plague cases. A general public health framework, including laws for plague control, decrees, institutions, and infrastructures was created. A system of basic welfare guaranteed by the city government was also established to satisfy the population needs in terms of medical treatment and food supplies and to implement disinfection of the houses (*25*). The pharmacists had to provide the poorest citizens with the necessary treatments (such as the Armenian bole, a ferruginous, ochreish, red clay used as a therapeutic substance against plague and all types of poisons). A list of the supplied treatments and a list of the citizens had to be kept to distinguish between the poorer and the richer. Richer persons would pay for their treatments, and the city government would pay for paupers (Instruction XXII). The *Morbers* were compelled to completely disinfect the city, house after house. The darkest houses and those lacking aeration had to be whitewashed by painters who had survived the contagion. Bonfires had to be set all around. For the less suspected houses, windows were required to be kept open at all times, perfumes to be sprayed, and all surfaces washed with vinegar (Instructions LIII and XLIX). Movements of people and goods to and from the city were strictly controlled during the epidemics (*17*).

Angelerio’s instructions and measures facilitated interventions and changed the way in which local health officers were selected. The town was divided into 10 wards. Each ward was controlled by a Health Deputy, who was invested with full authority according to the new anti-epidemic health laws, and a Plague Guard (Instruction II). The Health Deputies and the *Morbers* gathered twice a day in the so-called “City House” to follow the course of the epidemic and to transmit the information to the Councilors who were assisted by the physicians (Instruction VI) (*17*).

Angelerio’s health policy emphasized disease prevention through isolation of persons infected with or suspected to be infected with plague (Instructions IV, V). Persons suspected to have plague were isolated at a center (*tancat*) (Instruction XVIII), whereas plague patients were housed at a *lazaretto* (Instructions XXXI). The main city hospital, the Sant’Antonio, also served as a *lazaretto* to isolate the plague patients. Guards ensured isolation of the above centers (Instruction XX).

Fire had to be set to mattresses, fittings, and furniture from all houses in which cases of plague were registered (Instruction VII). When a person was suspected of having died of plague, the physicians or surgeons had to check the corps to establish whether the deceased person actually had died of plague. If the cause of the death was indeed plague, the victim’s relatives carried the corpse in the courtyard or left it outside the door (Instruction X).

Plague victims were buried in secluded cemeteries within 6 hours after death; burying plague victims inside the churches was strictly forbidden. Long and deep trenches were excavated, and the corpses were covered with lime to not corrupt the air and release mephitic vapors (Instruction XXXII). Grave diggers were selected from among persons who had contracted, and survived, plague during a previous outbreak in another town. They lived separately from the rest of the community and far from the hospital and were not allowed to leave their houses unless a Health Deputy accompanied them (Instruction XII) (*17*).

Moreover, Angelerio introduced a new method for sterilizing clothes, textiles, and objects according to the miasmatic–contagionistic notions. Stoves/ovens similar to those used to cook the flat tiles (*rejolas*) were kept constantly lighted by an underlying fire (Instruction XXXIX). The stove’s chamber was filled with the presumed textiles and objects after they had been washed under the *Morbers*’ supervision (Instruction XLIV) (*17*).

The causative agent of plague and its vector (rat fleas) were not known scientifically until the end of the 19th century (*28*,*29*). However, Angelerio clearly recognized the role of disinfection in controlling plague with dry heat to eliminate the responsible agents (which he called the *malefica semina*, “bad seeds”). The miasmatic–contagionistic theory implied to Angelerio that miasmatic contagion was present wherever plague patients had been. Therefore, everything they had worn or touched, as well as the place they had stayed, had to be disinfected (*3*,*4*).

Through the introduction of dry heat, both the elimination of the plague bacillus (*Yersinia pestis*) and its vectors (the fleas) were guaranteed (*30*). In addition, the vectors’ elimination helped prevent the transfer of infected fleas among citizens. Angelerio’s intuition, which led to selection of painters and grave diggers from among persons who had already acquired and survived plague, anticipates the notion that semi-immunity to the bubonic form of plague may develop in long-term recovered patients (*31*).

During the plague outbreak in Sardinia during 1652–1657, Angelerio’s instructions were resumed (*20*,*32*). The only 17th-century plague tract used in Sardinia, the *Tratado Universal* (Universal Tract), written by Juan Núnez de Castro in 1648, specifically refers to Angelerio’s instructions. Following his instructions, Núnez de Castro ordered the establishment of sanitary cordons, quarantines, isolation centers for persons with and suspected of having plague and for convalescents, and disinfection of clothes and houses; he also ordered the following of the previously detailed rules for mortuary hygiene (*17*,*32*). Núnez de Castro’s booklet was reprinted in Cagliari in 1652 by the *Protomedicus* Antonio Galcerín with the following title: *Instruccíon de las Prevenciones que se Hande Disponer en Tiempo de Contagio* (Instruction of the Preventive Measure that Have to be Applied during the Period of Contagion) (*8*). This plague guide became the sole reference for the Sardinian health authorities during the 1652–1657 outbreak that ravaged the island (*8*,*13*,*14*).

In conclusion, Angelerio’s observations and well-organized public health services contained the epidemic in the city and halted its spread. His modern prophylactic and hygienic measures represent a successful innovation in the sanitary system of 16th-century Sardinia and in the Mediterranean area and attest to the extraordinary efforts of the city government to prevent the introduction and spread of contagion. The measures he introduced in Sardinia paved the way for subsequent generations of physicians and enabled them to manage plague epidemics more efficiently.
